# Characterization of three glutamate decarboxylases from *Bacillus* spp. for efficient γ-aminobutyric acid production

**DOI:** 10.1186/s12934-021-01646-8

**Published:** 2021-08-04

**Authors:** Lei Sun, Yingguo Bai, Xiu Zhang, Cheng Zhou, Jie Zhang, Xiaoyun Su, Huiying Luo, Bin Yao, Yuan Wang, Tao Tu

**Affiliations:** 1 State Key Laboratory of Animal Nutrition, Institute of Animal Sciences, Chinese Academy of Agricultural Sciences, Beijing, 100193 China; 2 Ningxia Key Laboratory for the Development and Application of Microbial Resources in Extreme Environments, North Minzu University, Yinchuan, 750021 China

**Keywords:** γ-Aminobutyric acid, Bioconversion, Glutamate decarboxylase, Biocatalyst

## Abstract

**Background:**

Gamma-aminobutyric acid (GABA) is an important bio-product used in pharmaceuticals and functional foods and as a precursor of the biodegradable plastic polyamide 4. Glutamate decarboxylase (GAD) converts l-glutamate (l-Glu) into GABA via decarboxylation. Compared with other methods, develop a bioconversion platform to produce GABA is of considerable interest for industrial use.

**Results:**

Three GAD genes were identified from three *Bacillus* strains and heterologously expressed in *Escherichia coli* BL21 (DE3). The optimal reaction temperature and pH values for three enzymes were 40 °C and 5.0, respectively. Of the GADs, GADZ11 had the highest catalytic efficiency towards l-Glu (2.19 mM^− 1^ s^− 1^). The engineered *E. coli* strain that expressed GADZ11 was used as a whole-cell biocatalyst for the production of GABA. After repeated use 14 times, the cells produced GABA with an average molar conversion rate of 98.6% within 14 h.

**Conclusions:**

Three recombinant GADs from *Bacillus* strains have been conducted functional identification. The engineered *E. coli* strain heterologous expressing GADZ1, GADZ11, and GADZ20 could accomplish the biosynthesis of l-Glu to GABA in a buffer-free reaction at a high l-Glu concentration. The novel engineered *E. coli* strain has the potential to be a cost-effective biotransformation platform for the industrial production of GABA.

**Supplementary Information:**

The online version contains supplementary material available at 10.1186/s12934-021-01646-8.

## Background

As a four-carbon and water-soluble non-protein amino acid, γ-aminobutyric acid (GABA) plays an important role as an inhibitory neurotransmitter in mammals and plants [1]. Owing to its many biological activities, including roles in hypotension, sedation, diuresis, sleep enhancement and memory improvement, and hormone regulation, GABA has widely been incorporated in functional foods and pharmaceuticals [2–6]. Furthermore, GABA can be converted into polyamide 4, also known as nylon 4, which is a linear polymer of 2-pyrrolidone that can be chemically synthesized from GABA [7]. Nylon 4 has excellent physical properties and is environmentally safe owing to its heat resistance (melting point, 260 °C) and biodegradability [8]. GABA also has promising applications in the chemical industry and in efforts being undertaken to protect the environment [9]. Thus, developing a process to enable highly efficient GABA production subsequently is a key focus area for its industrial application.

**Fig. 1:**
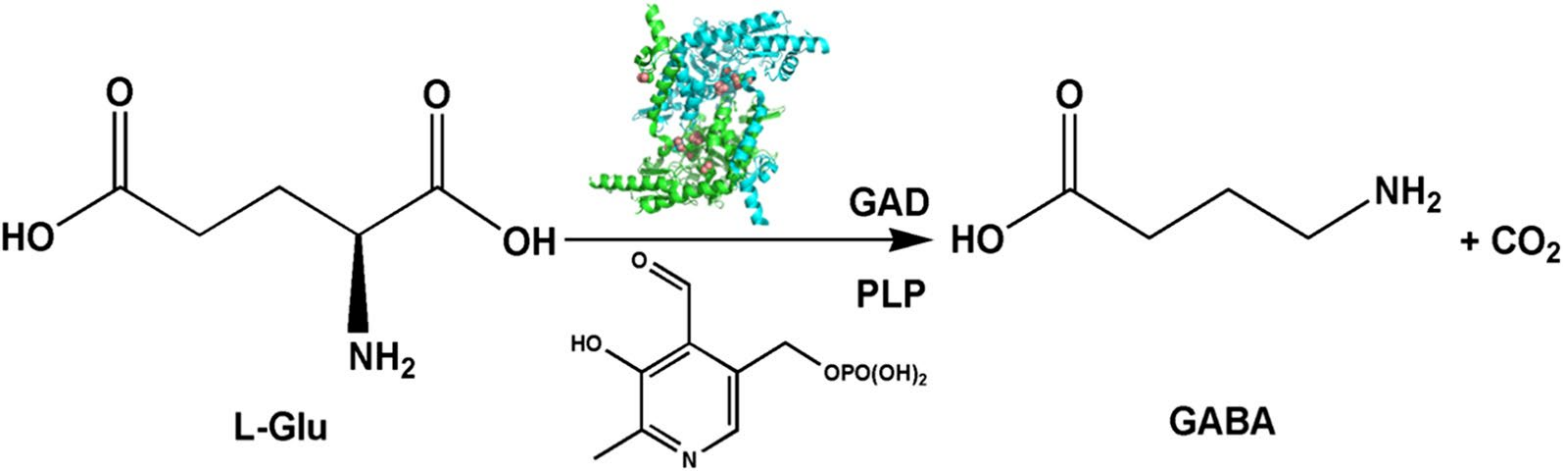
GABA production by glutamate decarboxylase (GAD) with pyridoxal-5′-phosphate (PLP) as a cofactor

GABA can be produced through the biocatalytic α-decarboxylation of l-Glu using glutamate decarboxylase (GAD, EC4.1.1.15) (Fig. 1) [10]. A variety of GADs are found in bacteria, actinomycetes, fungi, and plant, and they play a central role in the synthesis of GABA, using pyridoxal 5′-phosphate (PLP) as a cofactor. Generally, GABA biosynthesis is performed in the laboratory or commercially using isolated enzymes or whole cells [11]. Compared with those with using the purified enzyme method, several advantages have been observed in whole-cell bioconversion owing to the method’s high efficiency, simple preparation, and lower cost, which are of particular interest for large-scale, high-speed, industrialized GABA production processes [12]. GAD isolated from microbial sources, including *Escherichia coli*, *Bacillus megaterium*, lactic acid bacteria, and *Aspergillus oryzae*, are valuable for industrial production. The ability of GABA production varies between species and strains. As shown in Table 1, the yields of GABA production are varied from 5.26 to 614.15 g/L. Thus, it is important to explore strains expressing GADs with high catalytic capacity for the efficient production of GABA.

**Table 1:** GABA production by different microorganisms

Microorganism	Temperature (°C)	Time (h)	GABA production (g/L)	References
*Escherichia coli* BL21 (DE3)/GADZ11	37	1	103	This study
*Lactococcus lactis* FJNUGA01	30	6	98.4	[13]
*Chrysosporium lucknowense* C1	45	15	614.15	[14]
*Escherichia coli* W3110	50	72	12.37	[15]
*Lactobacillus brevis* NCL912	32	36	103.74	[16]
*Lactobacillus brevis* GABA 057	37	48	23	[17]
*Lactobacillus brevis*	45	10	201.8	[18]
*Streptococcus salivarius* ssp. *thermophilus* Y2	37	12	5.26	[19]

The genus *Bacillus* comprises gram-positive bacteria that are widely distributed on plant surfaces and in the soil and air. It produces many bioactive substances and various kinds of enzymes, such as amylases, proteases, and lipases [20]. As a result, the genomes of three *Bacillus* species, *Bacillus* sp. Z1, *Bacillus* sp. Z11, and *Bacillus* sp. Z20, were sequenced and analyzed. Each strain contained a putative *gad* gene, namely, *gadz1* in *Bacillus* sp. Z1, *gadz11* in *Bacillus* sp. Z11, and *gadz20* in *Bacillus* sp. Z20. The three relevant *gad* genes were cloned and expressed in *E. coli* BL21 (DE3). The target protein was purified for biochemical characterization using Ni–NTA chromatography. In addition, the capacity of the engineered *E. coli* BL21 (DE3) cells expressing GADZ1, GADZ11, and GADZ20 for GABA production was evaluated. Taken together, we have, here, presented an efficient biosynthetic pathway for the industrial production of GABA.

## Results

### Gene cloning and expression of putative GAD

**Fig. 2:**
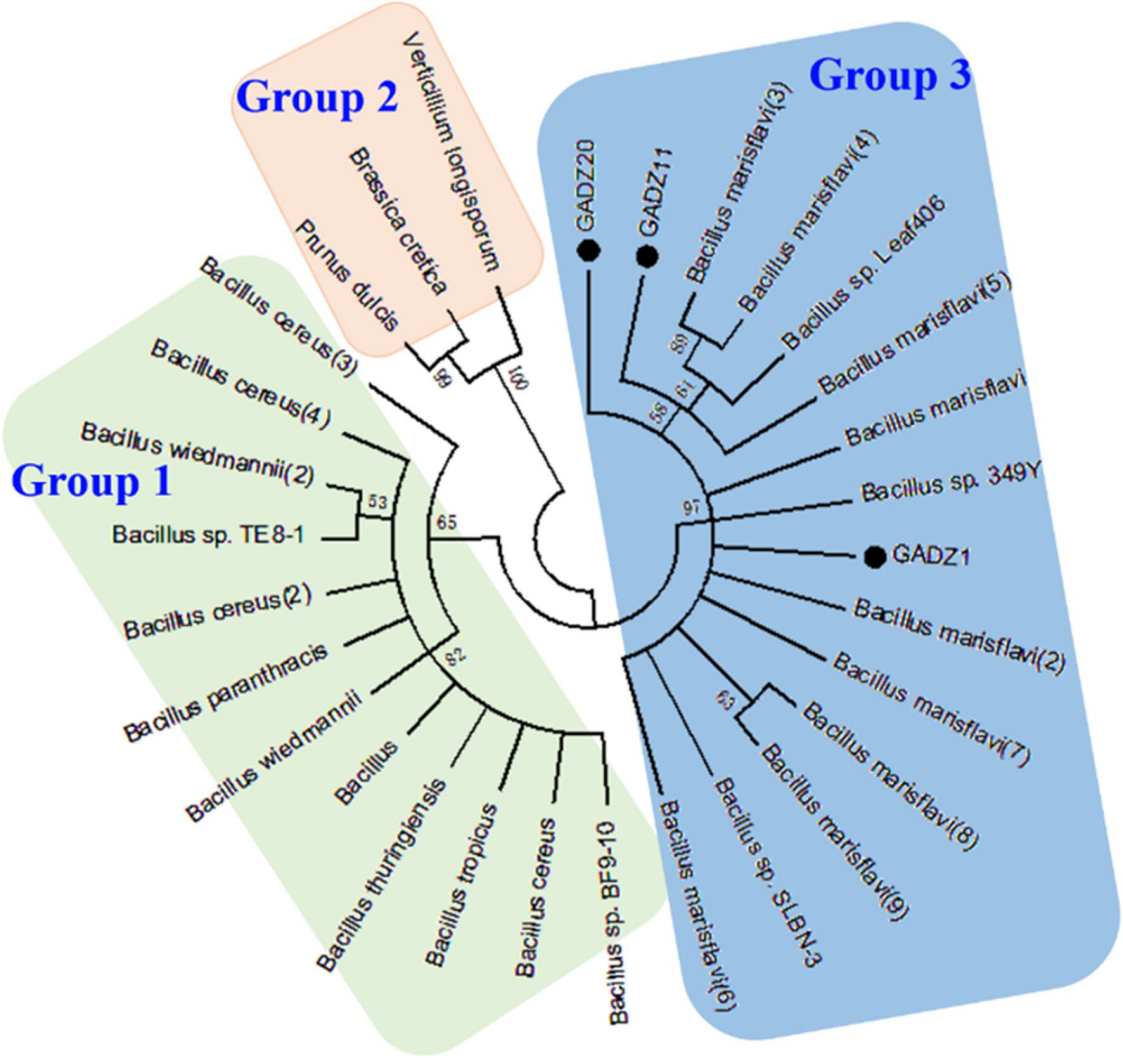
Phylogenetic tree of the GADs constructed using MEGA by the neighbor-joining method

The three GAD genes, *gadz1*, *gadz11*, and *gadz20*, were isolated from the corresponding *Bacillus* strains. The sequence length of the gad fragment was 1470 bp in all strains, and it encoded a 489-amino acids long polypeptide. Based on the sequence analysis, the three GADs were deduced to belong to the AAT–I superfamily and shared 96–97% sequence identities. Several conserved motifs were found; GETYTG (235–240) is probably the primary catalytic site or substrate-binding site, and the HVDAASGG (266–273) motif is highly conserved in PLP-dependent decarboxylases [21, 22]. Using the amino acid sequences of GADZ1, GADZ11, and GADZ20 as query sequences, a total of 27 homologous sequences were identified in the NCBI database. As shown in Fig. 2, phylogenetic analysis indicated that there were three branches of the GAD enzyme. GADZ1, GADZ11, and GADZ20 were located in the same clade, arising mainly from *Bacillus marisflavi* and *Bacillus* sp. 349Y. No putative signal peptide was predicted by using the SignalP-5.0 server, which indicated that the three GADs were intracellular enzymes. The calculated molecular masses were 55.5 kDa for GADZ1, 55.4 kDa for GADZ11, and 55.6 kDa for GADZ20. Further, the motif INVSGHKYGLVYPGLGWIIWR (295–315) is a PLP-binding domain, in which the ε-NH_2_ of Lys301 forms Schiff bases with the PLP cofactor through imine linkage. This step plays a key role in catalysis by the PLP-dependent decarboxylases [23, 24].

### Expression and purification of recombinant GAD

**Fig. 3:**
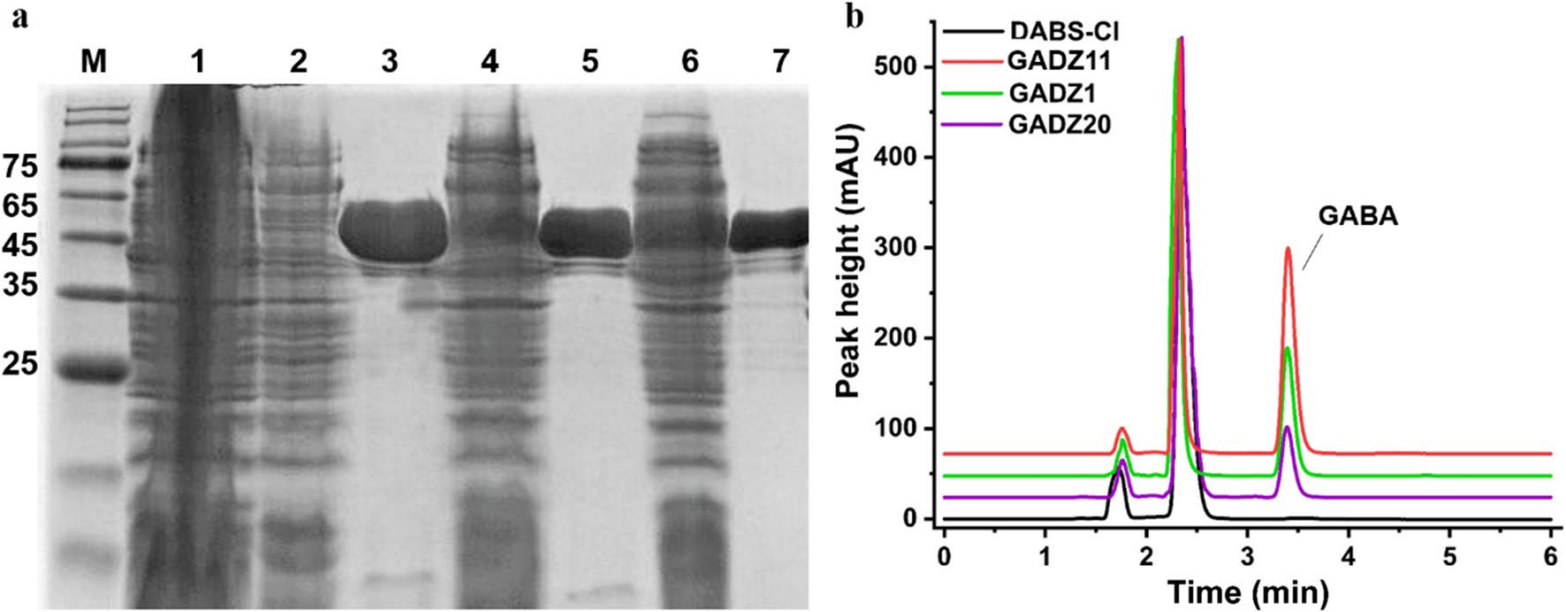
**a** SDS-PAGE analysis of purified recombinant GAD. M: protein ladder; 1: intracellular proteins of *E. coli* BL21 (DE3)/pET28a (+) (vector control); whole-cell intracellular proteins and affinity-purified proteins of *E. coli* BL21 (DE3)/GADZ1 (lanes 2 and 3), *E. coli* BL21 (DE3)/GADZ11 (lanes 4 and 5), and *E. coli* BL21 (DE3)/GADZ20 (lanes 6 and 7). **b** After incubation of the purified GADZ1, GADZ11, and GADZ20 proteins with l-Glu at pH 5.0, the reaction mixtures were analyzed using HPLC

The resulting plasmids, including pET28a-*gadz1*, pET28a-*gadz11*, and pET28a-*gadz20*, were successfully expressed in *E. coli* BL21 (DE3). Substantial GAD activity was detected after induction at 16 °C for 16 h. The three His6-tagged GADs were isolated using ultrasonic waves. A single Ni–NTA affinity chromatography step was used to purify the enzymes to > 95% purity, with an apparent molecular weight of 55 kDa (Fig. 3a). HPLC analysis revealed that GADZ11 had the best catalytic properties at pH 5.0 and 40 °C. The specific activity of the purified recombinant l-Glu enzymes towards l-Glu was 48.2 ± 1.5 U/mg for GADZ1, 98.9 ± 6.5 U/mg for GADZ11, and 13.5 ± 0.2 U/mg for GADZ20 (Fig. 3b).

### Functional identification of the three GADs

**Fig. 4:**
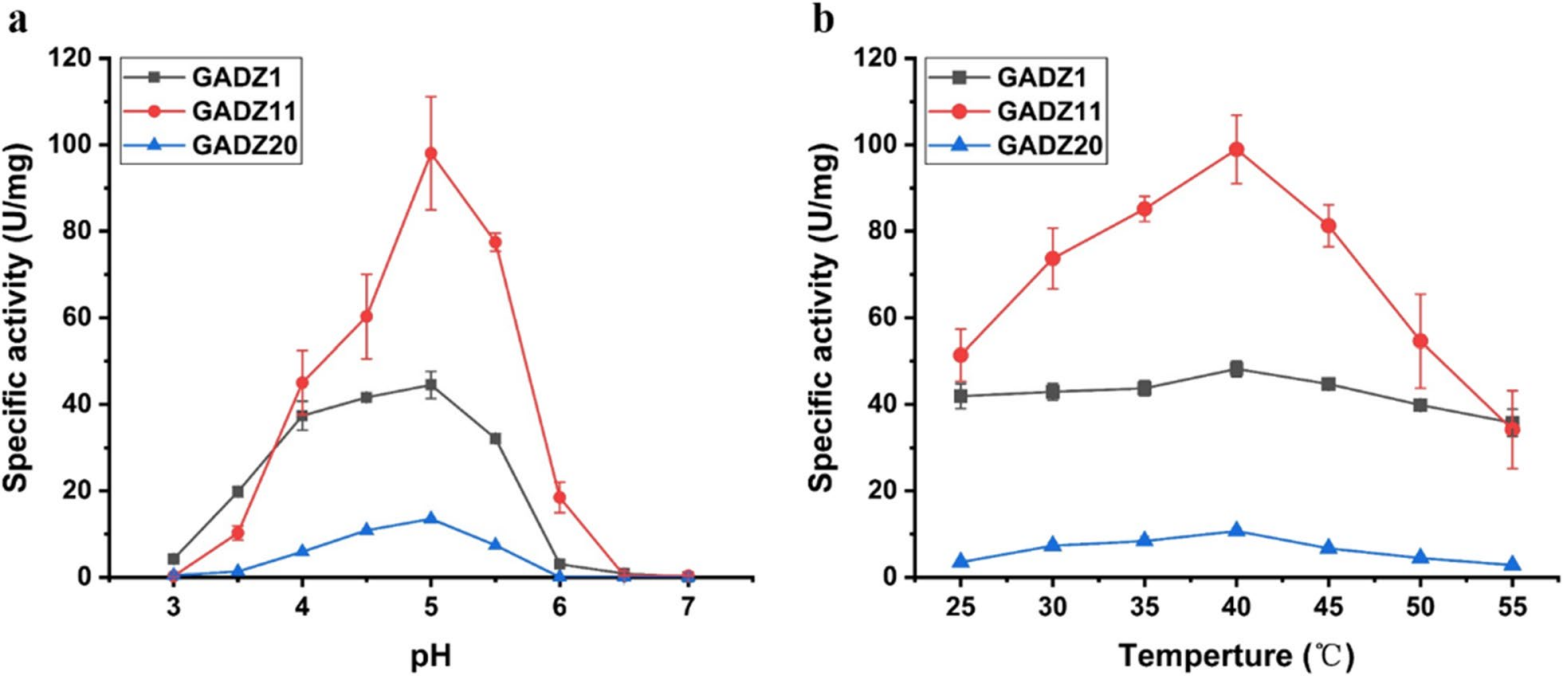
Enzymatic properties of GADZ1, GADZ11 and GADZ20. **a** The pH optima of GAD; **b** the temperature optima of GAD

The properties of the three purified GADs were compared with those of l-Glu. The three enzymes showed differences in catalytic activity. However, all enzymes exhibited the highest catalytic activity at pH 5.0 and 40 °C. Further, they retained 60% of the maximum activity in the pH range of 4.5–5.5 and more than 50% of the maximum activity in the temperature range of 25–50 °C. GADZ11 exhibited the best properties, and GADZ1 and GADZ20 exhibited only half and one-tenth of the activity of GADZ11, respectively (Fig. 4). The results indicated the potential value of GADZ11 in the bioconversion of l-Glu.

### The kinetic parameters of the three GADs

The kinetic parameters of the three GADs with reference to l-Glu were obtained and are summarized in Table 2. The purified GADZ11 enzyme showed a *k*_cat_ value that was ~ 2.1 and 8.4 times higher than that of purified GADZ1 and GADZ20, respectively, suggesting the high conversion efficiency of GADZ11. However, GADZ1 and GADZ20 exhibited low *K*_m_ values of ~ 13 and 75%, respectively, compared with that of GADZ11, suggesting the highest substrate-binding affinity of GADZ20. Based on these two aspects, the catalytic efficiency of GADZ11 (*k*_cat_/*K*_m_ values) was determined to be 2.19 mM^− 1^ s^− 1^, nearly 2-fold higher than that of GADZ1 and GADZ20. These results indicated that GADZ11 exhibited higher catalytic performance under the same reaction conditions than the other two GADs.

**Table 2:** Kinetic parameters of three GADs

Substrate	*V*_max_ (µMmin^−1^ mg^−1^)	*k*_cat_ (s^−1^)	*K*_m_ (mM)	*k*_cat_/*K*_m_ (mM^−1^ s^−1^)
GADZ1	58.02 ± 3.90	55.6 ± 5.88	45.40 ± 8.63	1.23
GADZ11	124.20 ± 4.83	114.86 ± 4.18	52.37 ± 5.44	2.19
GADZ20	14.75 ± 0.48	13.67 ± 0.33	13.26 ± 1.95	1.03

### Effect of different metal ions and EDTA on enzyme activities

Metal ions, like coenzymes, can change the catalytic activity of different enzymes in organisms and are often used as cofactors to promote or inhibit enzymatic reactions. The activity of GADZ11 was partially inhibited by administration of 1 mM of Ni^2+^ and EDTA, and more than 60% of the activity was retained. This was partially inhibited with the administration of 10 mM of Ni^2+^, K^+^, Cu^2+^, Ca^2+^, Mg^2+^, Fe^3+^, SDS, EDTA, and β-Mercaptoethanol, more than 40% of the activity was retained (Additional file 1: Figure S1). However, the presence of 1 mM SDS, 10 mM Ca^2+^, and EDTA significantly enhanced the enzyme activity by 60–80% for GADZ20. The addition of other reagents had little or no effect on enzyme activity.

### Whole cells bioconversion for GABA synthesis

GABA production efficiencies of the three *Bacillus* sp. strains and of *E. coli* recombinant strains were compared using l-Glu as substrate (Additional file 1: Figure S2). *E. coli* BL21(DE3)/GADZ11 exhibited 23-times higher GABA production than *Bacillus* sp. Z11. *E. coli* BL21(DE3)/GADZ1 and *E. coli* BL21(DE3)/GADZ20 exhibited decuple values higher than those of *Bacillus* sp. Z1 and Z20 strains, respectively. The results indicated that the three relevant *gad* genes achieved overexpression in *E. coli*.

**Fig. 5:**
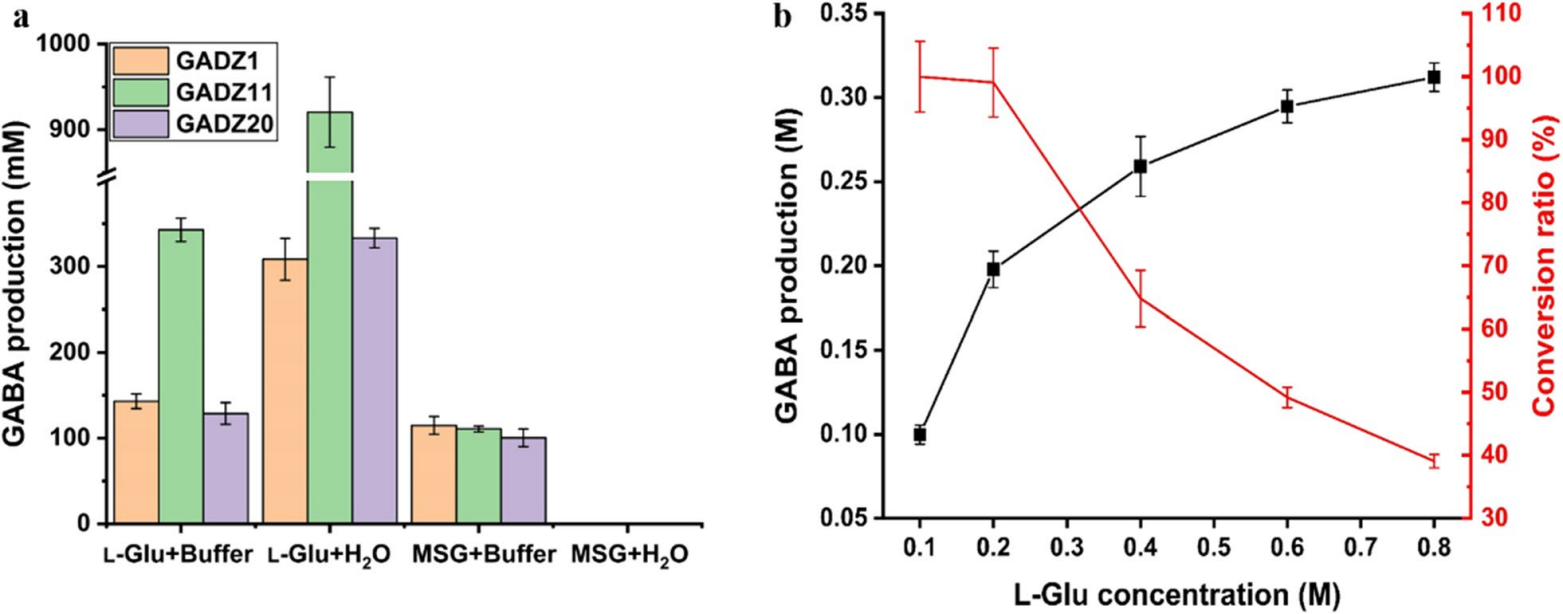
Biotransformation of *E. coli* to produce GABA. **a** Determining the suitable substrate (MSG/l-Glu) and reaction environment (buffer/water) for the conversion. **b** Conversion rate with different concentrations of l-Glu. The operating conditions chosen were as follows: reaction temperature, 37 °C; reaction time, 1 h; and PLP concentration, 0.02 mM. Data are shown as the mean ± standard deviation (n = 3)

In general, GAD can utilize both monosodium glutamate (MSG) and l-Glu as substrates [25]. To investigate the application value of the engineered *E. coli* strains expressing GADs in the context of GABA synthesis, whole-cell biosynthesis of GABA via l-Glu and MSG was performed. As shown in Fig. 5a, in both the sodium acetate solution and water, l-Glu and not MSG, was identified as the better substrate for producing GABA, irrespective of the presence of GAD-harboring *E. coli* strains. Nonetheless, the *E. coli* BL21(DE3)/GADZ11 strain was the best biocatalyst; the GABA yield using this strain with l-Glu was 343 ± 11 mM in sodium acetate buffer and 920 ± 33 mM in water. However, only 115 ± 8 mM GABA was produced in the sodium acetate buffer using the same strain with MSG. Thus, GABA production in the buffer was much lower than that in water, even when l-Glu was used as the substrate. The pH value was 4.6 at the beginning of the reaction mixture in the sodium acetate buffer, but it changed to 6.5 after a 1-h reaction. It is worth noting that no GABA presence was detected in the MSG reaction mixture when water was used. This proved that the reaction mixture was alkaline, and that an acidic environment was necessary for the biotransformation. Accordingly, the following whole-cell bioconversion studies were conducted in water reaction systems with l-Glu as the substrate.

To optimize GABA production, we explored the impact of the initial concentration of substrate in the reaction mix. The conversion rate was 99–100% when the concentration of l-Glu was lower than 1 M in the reaction mixture (Fig. 5b). When the l-Glu concentration was higher than 1 M, the conversion rate decreased substantially, though the yields of GABA were higher. We assumed that the higher the GABA concentration, the stronger the substrate inhibition. In addition, high concentrations of GABA can cause an osmotic pressure imbalance between the intracellular and extracellular environments; the resulting high extracellular osmotic pressure may negatively affect the conversion rate [26]. Thus, for the subsequent whole-cell bioconversion studies, 1 M l-Glu was chosen as substrate.

### The effect of cell concentration and PLP concentration on the conversion rate

**Fig. 6:**
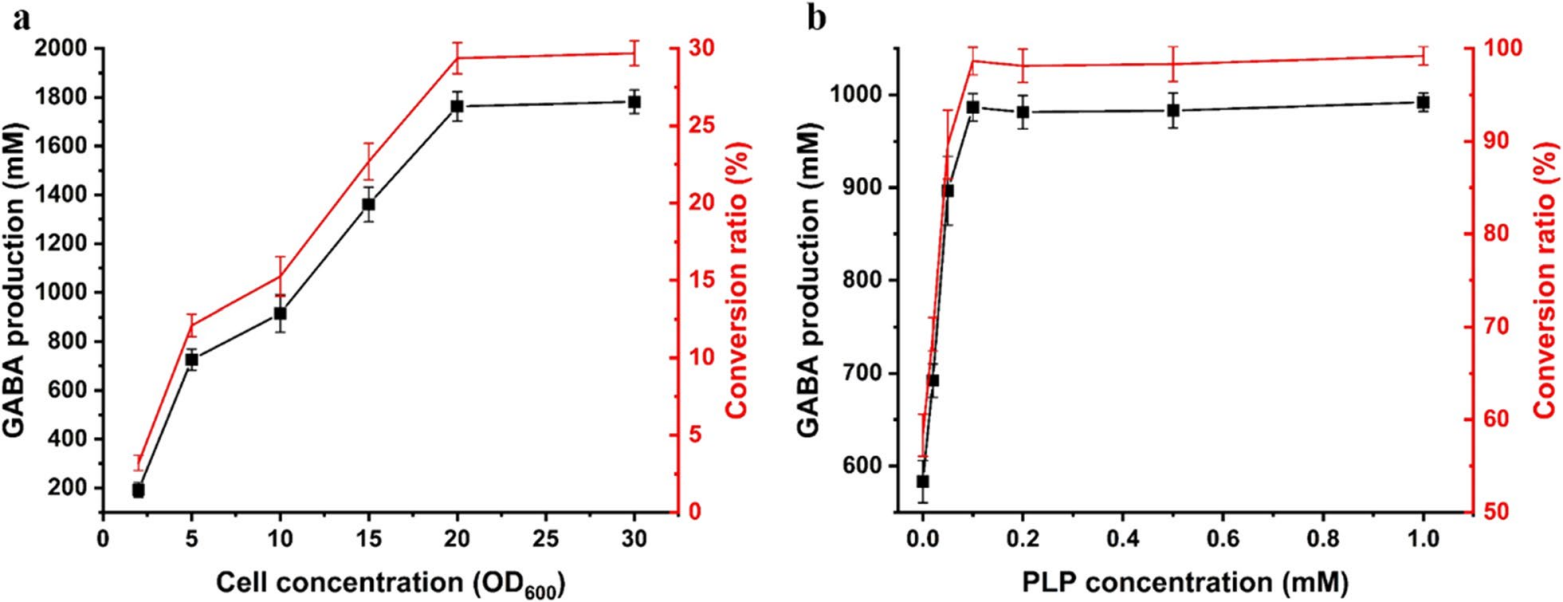
The optimum cell (**a**) and PLP (**b**) concentration for GABA production by *E. coli* BL21 (DE3)/GADZ11 in presence of l-Glu. The black line represents the yield of GABA and the red line indicates the conversion rate

The effect of different cell concentrations in the reaction system was examined at 37 °C for 2 h. To ensure that there was sufficient l-Glu for high cell concentration, we used 6 M l-Glu for GABA production. When the cell concentration was lower than 20 OD_600_, the conversion rate increased, thereby increasing cell concentration. When the cell concentration was higher than 20 OD_600_, the highest conversion rate was observed, with a GABA yield of 1.8 M (Fig. 6a). Thus, the cell concentration for subsequent studies was set at 20 OD_600_.

PLP was involved in the regulation of proton translocation required to catalyzes the decarboxylation of l-Glu, and the effect of different concentrations of PLP on GABA production was evaluated [27]. The GABA yield was 580 ± 18 mM without the addition of PLP, and it did not change with increasing amounts of PLP supplementation beyond 0.1 mM PLP (Fig. 6b). Thus, when using 1 M l-Glu as substrate, the optimal PLP concentration to be added was determined to be 0.1 mM.

### The upper bound estimation of *E. coli* BL21(DE3)/GADZ11 to produce GABA from l-Glu

**Fig. 7:**
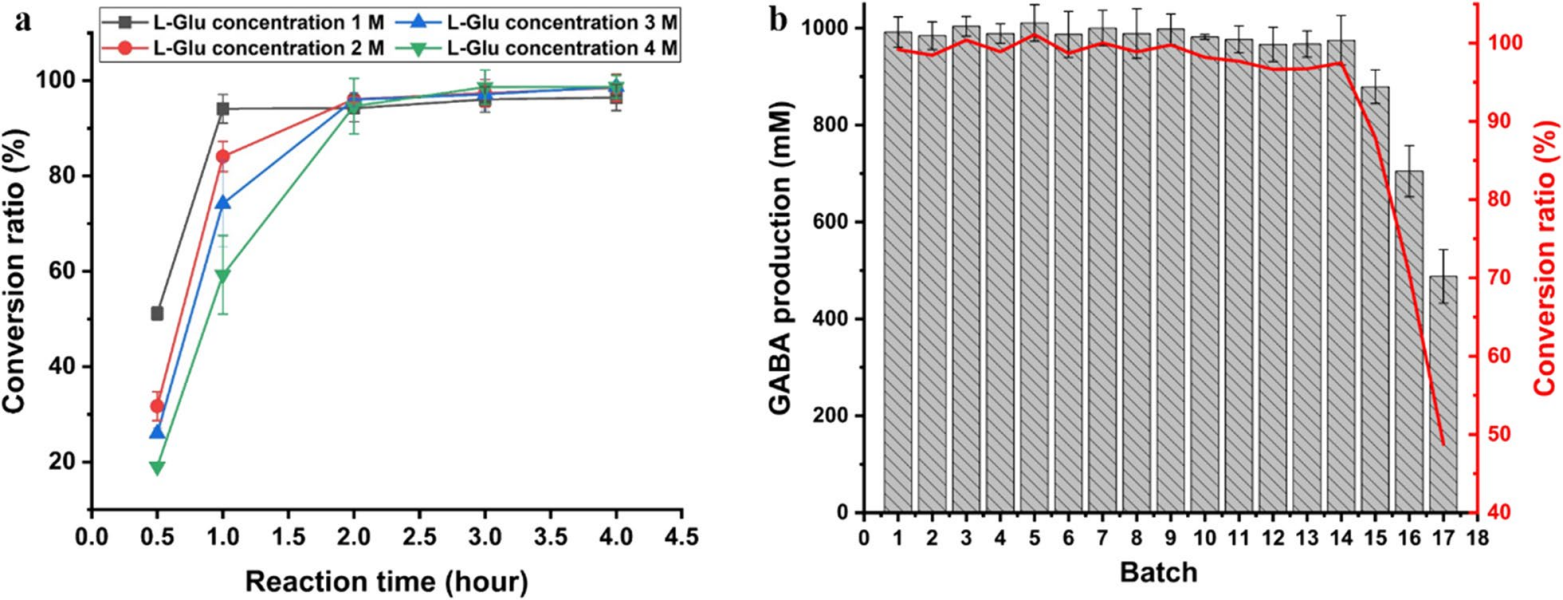
Upper bound estimation of conversion by *E. coli* BL21(DE3)/GADZ11 from l-Glu. **a** Time-history analysis of GABA formation in single-batch reactions with different molar concentrations of l-Glu. **b** Reused of *E. coli* BL21(DE3)/GADZ11. Conditions for each batch condition were as follows: 1 h, 1 M l-Glu, and 0.1 mM PLP

Next, we tested the time profiles of GABA production with the use of different concentrations of l-Glu (Fig. 7a). *E. coli* BL21(DE3)/GADZ11 could convert 94% of 1 M l-Glu to GABA in 1 h, while for 2 M l-Glu, complete conversion was achieved in 2 h. The highest efficiency of GABA production was observed in the first hour of the reaction. In this first hour, 941 ± 25 mM (94.1%), 1682 ± 58 mM (84.1%), 2226 ± 221 mM (74.2%), and 2371 ± 269 mM (59.3%) GABA was produced from 1 to 4 M l-Glu, respectively. Based on the conversion rate, the optimal addition rate of l-Glu was determined to be 1 M for the batch reactions.

Determining whether cells are capable of efficient recovery and reuse is of great significance in reducing production costs (Fig. 7b). In this study, the conversion ratio of l-Glu to GABA was 99 mol% in the first hour, and almost complete conversion was achieved in the subsequent 13 reaction batches. Each reaction could reach this rate with the use of 1 M l-Glu and 0.1 mM PLP. Finally, *E. coli* BL21(DE3)/GADZ11 could be used for 14 batch conversions, with a conversion rate of 96–100%. Within 14 h, 14 M l-Glu was converted to 13.8 M GABA, with an average molar conversion rate of 98.6%. After 14 batch conversions, the conversion ratio was gradually decreased to 48.7% until 17 batch conversions were executed. The accumulated yield of GABA was 15.9 M (equal to 1.64 kg/L) from a total of 17 M of l-Glu (equal to 2.5 kg/L). Thus, *E. coli* BL21(DE3)/GADZ11 showed potential as a strain with excellent conversion properties.

### Purification and crystallization of GABA

**Fig. 8:**
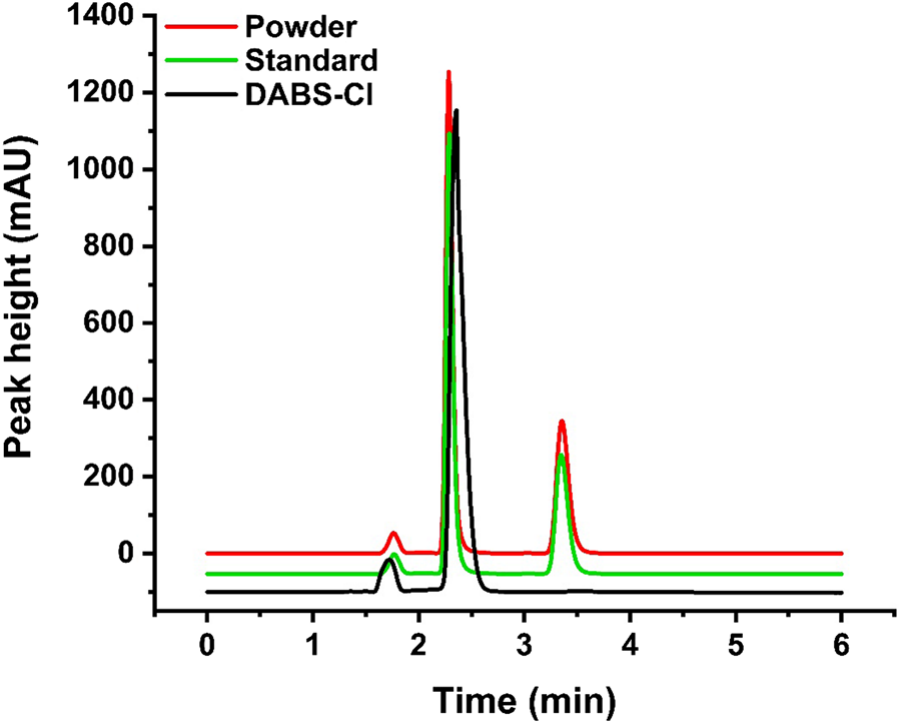
The sample and reference standard were analyzed by HPLC

In previous batch studies, 14 M l-Glu was completely converted over 14 h to produce 13.8 M GABA in a 280 mL water system. The reaction mixture was collected by centrifugation and concentrated by rotary evaporation. The synthesized GABA was dried to a white powder at 65 °C. The GABA powder thus obtained was highly pure, as shown in the HPLC chromatogram (Fig. 8). There was no difference between the sample and the reference standard with respect to purity.

## Discussion

In recent years, GABA has become hugely popular as a bioactive component in the pharmaceutical and food industries. At present, global GABA trade revenue is approximately 77 million dollars, and it is expected to reach 96.4 million dollars in 2026, with a compound annual growth rate of 5.9%. In China, the total market value of GABA and its related products is about 2.5–3.0 billion yuan with a predicted of steady growth [28, 29]. Thus, the development of a reliable and stable platform for the cheap production of GABA is of great economic significance. With the intent of developing a novel biotransformation system, we identified three GADs from three *Bacillus* spp. that had similar optimum pH values and temperatures as those of other strains. Of note, the catalytic efficiency of the purified GADZ11 was 2.19 mM^− 1^ s^− 1^, higher than that in previous reports on GAD (Table 3). The sequence similarity between GADZ1, GADZ11, and GADZ20 was 97.6–98.6% (Additional file 1: Figure S3; 24 residues were different). However, there was significant activity variation among them (Fig. 4). Huang et al. [30] demonstrated that residues (Ser126, Ser127, Cys168, Ile211, Ser276, His278, and Ser321) in GAD from *Lactobacillus brevis* CGMCC 1306 play important roles in anchoring the PLP cofactor to the active site, thereby supporting its catalytic reactivity. Tavakoli et al. [31] identified, through a stimulated docking study, that performing mutations separately at positions Ile164, Asn302, Asp304, Tyr393, Ser396, Arg398, and Thr410 could increase binding affinity to substrate. The corresponding residues in GADZ11 are Asp126, Cys127, Arg164, Ala168, His211, Ala276, Phe278, Tyr302, Leu304, Pro321, Asn393, Asp396, Pro398, and Ser410, which are totally different to those in the two enzymes mentioned above. In our future studies, structural crystallization and site-directed mutagenesis would be employed to reveal the mechanism underlying the high catalytic activity of GADZ11. Furthermore, the *E. coli* cells heterologously expressing GADZ11 could produce GABA directly using high-concentration l-Glu in a buffer-free reaction, with conversion rate was over 95%. These excellent transformation properties are more suited for the commercial production and post-purification of GABA.

**Table 3:** Essential properties and kinetic parameters of GADs in this study and other microbial GADs

Microorganism	pH optimum	Temperature optimum (°C)	*k*_*cat*_/*K*_m_ (mM^-1^ s^-1^)^a^	References
*Bacillus* sp. Z11	5.0	40	2.19	This study
*Bacillus* sp. Z1	5.0	40	1.23	This study
*Bacillus* sp. Z20	5.0	40	1.03	This study
*Lactiplantibacillus plantarum*	5.0	40	0.00012	[32]
*Lactobacillus brevis* 877G	4.2	45	0.7	[33]
*Lactobacillus paracasei*	5.0	50	1.43	[34]
*Lactococcus lactis*	4.7	30	ND	[35]
*Streptomyces chromofuscus*	4.2	37	1.21	[36]

*ND* not determined

^a^Catalytic efficiency (*k*_cat_/*K*_m_ value) was determined using l-Glu as the substrate

To date, most reported GADs are more active and stable within the temperature and pH ranges of 30–50 °C and 4.0–5.0, respectively, and their activities drop rapidly at pH > 6 [37]. In microorganisms, GAD activity is an important mechanisms of resistance to low-acid environments, which is why most GADs only work at a low pH, including the GADZ11 used in our study [38]. The low pH range (pH 4–5) of the reaction buffer is a critical limitation for the efficient production of GABA [39]. GABA conversion by *E. coli* BL21(DE3)/GADZ11 reached 34.3% in sodium acetate solution and 92.0% in water. Using MSG, the conversion rate was 11.1% in sodium acetate buffer and almost nil in water. These results explain why MSG is not the best substrate choice for GABA production [40, 41]. The pH of a solution containing 1 M MSG in 20 mL of water is pH 7.0, while that of a solution containing the same concentration of l-Glu in water is 2.3. Moreover, l-Glu is less soluble in water than MSG. The solubility of l-Glu in water is 15.1 g/L at 40 °C, while that of MSG is 717 g/L. Therefore, most of the l-Glu remains in the reaction system in solid form. In other words, the osmotic pressure of the mixture with l-Glu is much lower than that of the mixture with MSG, a feature that favors the biosynthesis of GABA. Furthermore, when the concentrations of l-Glu and MSG were equal in the reaction system, l-Glu could maintain an acidic pH while being consumed [12, 42]. Thus, l-Glu is a better choice for the synthesis of GABA.

It is known that GAD is a typical PLP-dependent enzyme [26]. PLP is a cofactor for a variety of enzymes involved in the metabolism of amino compounds and the synthesis of biomolecules, such as dopamine, epinephrine, norepinephrine, and histamine [43]. In our study, PLP did not seem to be necessary for the biosynthesis of GABA, with the production of GABA at 583 ± 23 mM without the addition of PLP to the 1 M l-Glu sustrate. However, it is difficult to determine whether PLP exists naturally in *E. coli*. Although it was not necessary in our system, the addition of PLP has previously demonstrated substantial positive effects on GABA production [36]. Our results are consistent with those of other studies. The rate of conversion increased with increase in PLP concentration and the maximum production achieved was 985 ± 15 mM (98.5%).

Our study revealed that the conversion of 4 M l-Glu required 2 h to reach 95 mol%, with a cell concentration of 20 OD_600_ and PLP concentration of 0.4 mM. Therefore, the consideration of time notwithstanding, given proper concentrations of cells and PLP, l-Glu can be completely converted to GABA in a short period. Furthermore, high purity of GABA can be obtained by simple purification and concentration from the reaction mixture. To summary, the platform that we developed has the following merits: the *E. coli* strain engineered in this study may be used in industry for the commercial-scale production of GABA, and our results provide the preliminary data for the separation and purification of GABA from fermentation broth.

## Conclusions

Functional identification was conducted for three recombinant GADs from *Bacillus* strains. Of them, GADZ11 possessed the best bioconversion properties. An engineered *E. coli* strain heterologously expressing GADZ1, GADZ11, and GADZ20 could accomplish the biosynthesis of l-Glu to GABA in a buffer-free reaction with a high l-Glu substrate concentration. The engineered *E. coli* BL21 (DE3)/GADZ11 strain was able to achieve the complete conversion of 1 M l-Glu in 1 h. We believe that the novel engineered *E. coli* strain has the potential to be a cost-effective biotransformation platform for the industrial production of GABA.

## Methods

### Strains, media, plasmids, and chemicals

The three *Bacillus* spp. were isolated from desert sand samples obtained from the Ningxia Province, China, and deposited in the Agricultural Culture Collection of China under registration numbers ACCC 61750, 61747, and 61748. These strains were cultured in Luria–Bertani medium at 30 °C. *E. coli* XL10 was used for gene cloning. *E. coli* BL21 and plasmid pET-28a were used as the expression host and vector, respectively. A DNA purification kit, LA Taq DNA polymerase, and restriction endonucleases were purchased from TaKaRa (Tsu, Japan). T4 DNA ligase was purchased from New England Biolabs (Hitchin, UK). All chemicals were of analytical grade and were commercially available.

### Cloning of *gad* genes from *Bacillus* strains and plasmid construction

Genomic DNA from *Bacillus* strains grown in LB medium was extracted using the Wizard Genomic DNA Purification Kit (Promega, Madison, WI, USA). The GAD genes, *gadz1*, *gadz11*, and *gadz20* (GeneBank accession numbers MW703457, MW703456 and MW703455) were amplified by PCR from the genomic DNA of *Bacillus* strains using suitable primer pairs (GADF: 5′-CTGAATTCATGTCCAAGGATCGAAAAGCAG-3′ and GADR: 5′-TTCGCCGGCGAAGCGGCCGCCTAATGATGAAACCCATT-3′). The amplified DNA fragment was purified from a 1.0% agarose gel using the Wizard SV Gel and PCR Clean-Up System (Promega) after gel electrophoresis. The purified 1470 bp gad fragment was digested with *EcoR*I and *Not*I and ligated (T4 DNA ligase) into pET-28a (+) to generate pET-28a-*gadz1*, pET-28a-*gadz11*, and pET-28a-*gadz20*. The constructed plasmids were used for expression in *E. coli* BL21 (DE3).

### Expression and enzyme purification

*E. coli* BL21(DE3) transformed with the plasmids pET-28a-*gadz1*, pET-28a-*gadz11*, and pET-28a-*gadz20* were cultured in LB media containing kanamycin (50 µg/mL) at 37 °C for 12 h. Then, the culture was transferred to 400 mL of LB broth at 37 °C (1% by volume of inoculant). When suitable bacterial concentration was achieved (OD_600_ of 0.6–0.8), protein expression was induced by adding 1 mM isopropyl β-d-1-thiogalactopyranoside (IPTG) and shaking at 200 rpm (16 °C, 16 h). The cells were collected at 8000*×g* for 10 min and resuspended in lysis buffer (20 mM Tris-HCl buffer, 500 mM NaCl, pH 7.6). Then, cells were disrupted by ultrasonic waves. After centrifugation, the protein was separated by Ni–NTA affinity chromatography using an elution buffer (20 mM Tris-HCl buffer, 500 mM NaCl, 200 mM imidazole, pH 7.6), then the proteins in the supernatant and pellet were resolved through SDS-PAGE [44]. Final protein concentrations were determined using the Bradford assay (BSA was used as a standard) [45].

### Determination of enzyme activity and GABA formation

Enzyme activity was determined by measuring GABA production rate using HPLC analysis, with some modifications [46, 47]. The reaction mixture comprised 400 µL of Na_2_HPO_4_-citric acid buffer (80 mM, pH 6.0), 500 µL of l-Glu (50 mM), 50 µL of PLP (0.02 mM), and 50 µL of purified enzyme. Ice-chilled 80% ethanol was added in an equal volume (1 mL) to stop the reaction after 30 min at 40 °C. The reaction supernatant (500 µL) was mixed with 100 µL of NaHCO_3_ (2.5 g/L) and 200 µL of 4-*N*, *N*-dimethylaminoazobenzene-4′-sulfonyl chloride (DABS-Cl) (0.25 g/L, dissolved in acetonitrile), and incubated at 70 °C for 20 min. This was followed by analysis using a SHIMADZU 20 A series instrument (Shimadzu, Kyoto, Japan) and an Agilent ZORBAX SB-C18 column (5 μm, 4.6 × 150 mm) (Agilent, Santa Clara, CA, USA). The mobile phase was a solution of 35% (v/v) acetonitrile solution and 65% 50 mM sodium acetate. The flow rate and column temperature were 1 mL/min and 30 °C, respectively; the injection volume was 10 µL; and the detection wavelength was 436 nm. The GABA content in the test solutions was calculated by taking into consideration the peak areas observed with the standard. One enzyme activity unit was defined as the amount of enzyme required for the release of 1 mM free GABA in 1 min. Three parallel wells were set up per group.

### Optimum pH and temperature assay

The optimum pH properties of the GADs were determined at reaction pH values from 3.0 to 7.0. pH-activity profiles were examined for 30 min at 37 °C. Optimum temperature was examined at pH 5.0 over a temperature range of 25–55 °C. Each experiment was performed in triplicate.

### Enzyme kinetic assays

Enzyme kinetic assays were performed in Na_2_HPO_4_-citric acid buffer containing 5–150 mM l-Glu at 40 °C for 15 min. The pH of the mixture containing l-Glu and Na_2_HPO_4_-citric acid buffer was 5.0. Each experiment was performed in triplicate. The *K*_m_ and *V*_max_ values were nonlinearly fitted using the GraphPad Prism 5 software.

### Effect of metal ions and chemical reagents on enzyme activity

To determine their effects on the enzyme activity of GAD, various metal ions (Na^+^, Ni^2+^, K^+^, Cu^2+^, Co^2+^, Ca^2+^, Mg^2+^, and Fe^3+^) and chemical reagents (SDS, EDTA, and β-Mercaptoethanol) were added into the reaction system individually, with their final concentration maintained at 1 mM and 10 mM, respectively. Then, the residual enzyme activity was determined under standard conditions. The system without any additives was used as the control.

### Whole-cell bioconversion process

Recombinant *E. coli* cells harboring GADZ1, GADZ11, and GADZ20 were cultured at 37 °C. Then, protein expression in the engineered bacteria was induced at 16 °C for 16 h. After this, the cells were centrifuged at 8000*×g* for 10 min, washed, and resuspended in water containing MSG or l-Glu at appropriate concentrations. OD_600_ was measured to indicate cell concentration. The reaction of the 20 mL mixture in 100 mL flasks was performed at 37 °C at 120 rpm. GABA production as a result of the reaction was analyzed and the rate was evaluated by HPLC.

To optimize the reaction conditions, the effects of substrate specificity, substrate concentration, cell concentration, PLP concentration, time-course analysis of single-batch reactions, and the recycling ratio of batch reactions were analyzed simultaneously in this study.

### Substrate specificity and optimum substrate concentration

The substrate specificities of whole-cell biosynthesis were investigated in an assay system containing the following substrates: MSG or l-Glu. The whole-cell biotransformation reactions were conducted in 0.1 M sodium acetate buffer (pH 4.6) or water as part of by single factor and orthogonal experiments. The reaction conditions were designated as follows: reaction time, 1 h; OD_600_, 20; and l-Glu or MSG concentration, 1 M.

To determine the impact of the l-Glu concentration on GABA production, the following procedure was performed: after 16 h preculture, *E. coli* BL21(DE3)/GADZ11 cells were centrifuged and suspended in water. The reaction system, thus, consisted of 20 mL of water containing resuspended cells in a 100 mL Erlenmeyer flask. To this, different amounts of l-Glu (0.5, 1, 2, 3, and 4 M) were added. The reaction mixture contained 0.02 mM PLP. The reaction was stopped by adding 30 mL of ethanol after 1 h, and the volume was made up to 100 mL with water. The reaction supernatants were collected to measure the GABA content.

### Optimum cell and PLP concentration

To determine the optimal cell concentration required for the conversion, the following procedure was performed: the 20 mL reaction system was supplemented with 6 M l-Glu, 0.02 mM PLP, and a predetermined quantity of cells (OD_600_ 2, 5, 10, 15, 20, 30). This reaction mixture was incubated in a shaker at a specified shaking rate (120 rpm) at 37 °C for 2 h. Aliquots (500 µL) were withdrawn into an equal volume of ice-chilled 80% ethanol after 2 h to stop the reaction. The reaction supernatants were collected to measure the content of GABA via HPLC.

To determine the effects of the coenzyme PLP on the reaction, 0 (control), 0.02, 0.05, 0.1, 0.2, 0.5, and 1 mM PLP was added to the 20 mL mixture. The l-Glu and cell concentrations were 1 M and 20 OD_600_, respectively. After 2 h of culture at 37 °C, 500 µL of the reaction mixtures were taken and measured using HPLC.

### The upper bound estimation of *E. coli* BL21 (DE3)/GADZ11 to convert GABA from l-Glu

To determine the optimal reaction conditions, and to thereby decrease manufacturing cost, reactions with different concentrations of l-Glu were conducted for the time-course assays. PLP at a final concentration of 0.4 mM and cells at a final concentration of 20 OD_600_ were added to the reaction system, and the l-Glu concentrations tested were 1, 2, 3, and 4 M. To stop the reaction, 30 mL of ethanol was added to the mixtures (when the solid l-Glu was fully consumed), and water was added to make up the volume to 100 mL. The reaction mixtures were subjected to HPLC.

The concentrations of l-Glu and PLP were 1 M and 0.1 mM, respectively, and the cell concentration was 20 OD_600_ in the batch reaction. The reaction was conducted at 37 °C for 1 h, and the cells were collected by centrifugation for the next batch when the solid l-Glu was fully consumed. There were 17 batches in total. The samples were tested when the reactions were complete.

### Conversion of high concentrations of l-Glu and the purification and crystallization of GABA

To prepare GABA crystals, the mixture from the previous eight batches was separated and concentrated by rotary evaporation. GABA crystals were collected from the concentrated solution. The crystals were oven-dried at 65 °C until they were converted into a white powder [48]. The purity of the powder was determined by HPLC.

## Supplementary Information

**Additional file 1:** **FigureS1.** The effect of metal ions and chemical reagents(1 mM and 10 mM) on enzyme activity**. FigureS2.** The comparison of GABA production efficiencies between the three *Bacillus* sp. strains and of *E. coli*recombinant strains. **FigureS3.** Multiple sequence alignment of GADZ1, GADZ11and GADZ20.

## Data Availability

All data generated or analyzed during this study are included in this published article and its additional files.

## Abbreviations

l-Glu: l-Glutamate
MSG: Monosodium glutamate
GABA: Gamma-aminobutyric acid
SDS-PAGE: Sodium dodecyl sulfate-polyacrylamide gel electrophoresis
GAD: Glutamate decarboxylase
*K*_m_: Michaelis constant
*V*_max_: Maximum reaction rate
*k*_cat_: Turnover number
PLP: Pyridoxal 5′-phosphate
DABS-Cl: 4-*N*, *N*-dimethylaminoazobenzene-4′-sulfonyl chloride
