# Supplementary material for: Characterization of three glutamate decarboxylases from Bacillus spp. for efficient γ-aminobutyric acid production
Source: Microb Cell Fact. 2021 Aug 4;20:153. doi: 10.1186/s12934-021-01646-8 (PMC8336373; doi:10.1186/s12934-021-01646-8)

**Additional Information**

**Characterization of three glutamate decarboxylases from *Bacillus* spp. for efficient γ-aminobutyric acid production**

Lei Sun^1^, Yingguo Bai^1^, Xiu Zhang^2^, Cheng Zhou^2^, Jie Zhang^1^, Xiaoyun Su^1^, Huiying Luo^1^, Bin Yao^1^, Yuan Wang^1,^*, Tao Tu^1,^*

*^1^*State Key Laboratory of Animal Nutrition, Institute of Animal Sciences, Chinese Academy of Agricultural Sciences, 100193 Beijing, China

*^2^*North Minzu University, Ningxia Key Laboratory for the Development and Application of Microbial Resources in Extreme Environments, 750021 Yinchuan, China

* Corresponding authors. State Key Laboratory of Animal Nutrition, Institute of Animal Sciences, Chinese Academy of Agricultural Sciences, 100193 Beijing, China. Tel.: +86 10 82106053; fax: +86 10 82106054.

*E-mail addresses*: [tutao@caas.cn](mailto:tutao@caas.cn); wangyuan08@caas.cn.

**Additional Figures**

**Figure S1** The effect of metal ions and chemical reagents (1 mM and 10 mM) on enzyme activity.


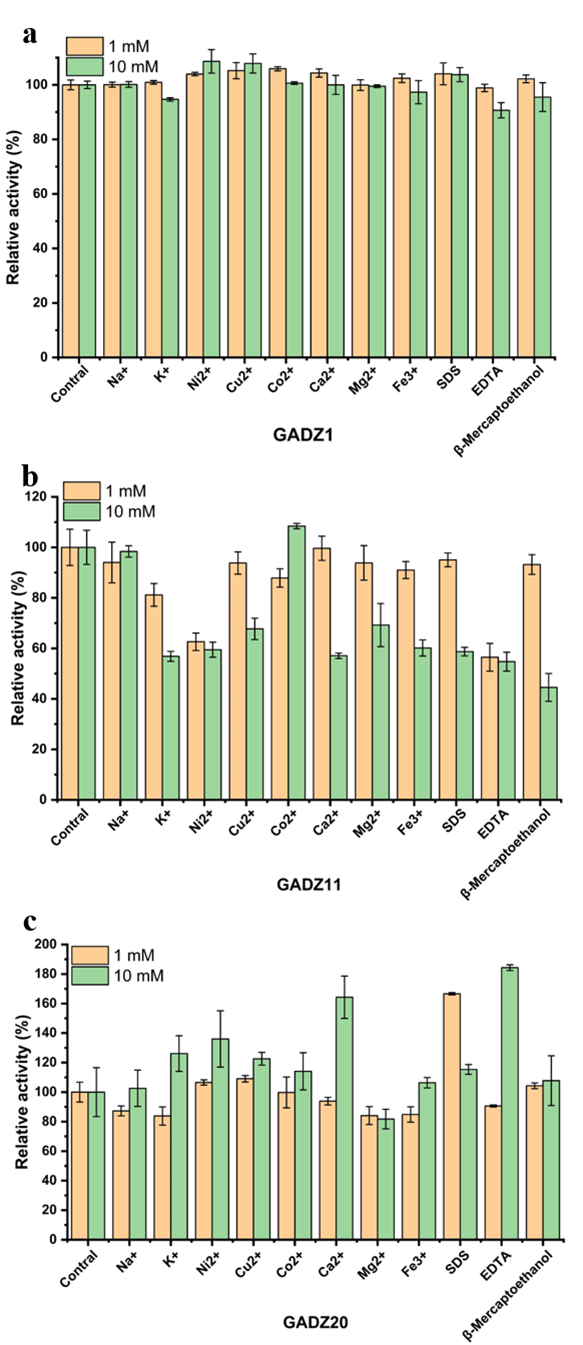


**Figure S2** The comparison of GABA production efficiencies between the three *Bacillus* sp. strains and of *E. coli* recombinant strains.


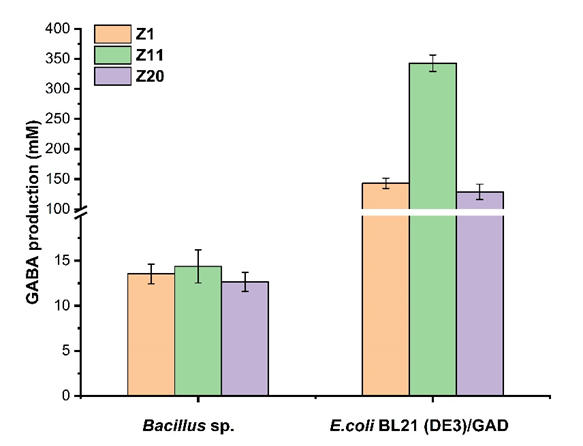


**Figure S3** Multiple sequence alignment of GADZ1, GADZ11 and GADZ20.


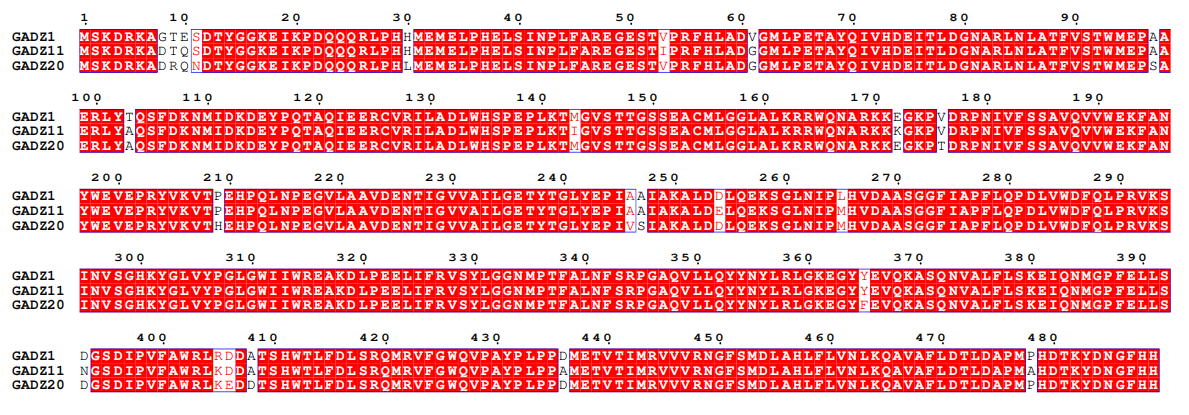

Supplement: Supplementary file 1 — Additional file 1: FigureS1. The effect of metal ions and chemical reagents(1 mM and 10 mM) on enzyme activity. FigureS2. The comparison of GABA production efficiencies between the three Bacillus sp. strains and of E. colirecombinant strains. FigureS3. Multiple sequence alignment of GADZ1, GADZ11and GADZ20. [file 12934_2021_1646_MOESM1_ESM.docx]
